# Noscapine Induced Apoptosis via Downregulation of Survivin in Human Neuroblastoma Cells Having Wild Type or Null p53

**DOI:** 10.1371/journal.pone.0040076

**Published:** 2012-07-26

**Authors:** Shiwang Li, Jing He, Shuai Li, Guoqing Cao, Shaotao Tang, Qiangsong Tong, Harish C. Joshi

**Affiliations:** 1 Department of Pediatric Surgery, Union Hospital, Tongji Medical College, Huazhong University of Science and Technology, Wuhan, China; 2 Institute of Hematology, Union Hospital, Tongji Medical College, Huazhong University of Science and Technology, Wuhan, China; 3 Department of Cell Biology, Emory University, Atlanta, Georgia, United States of America; Vanderbilt University Medical Center, United States of America

## Abstract

Neuroblastoma is the most common extracranial solid tumor of childhood. It accounts for 15% of pediatric cancer deaths. Chemotherapy is the mainstay of treatment in children with advanced neuroblastoma. Noscapine, a nontoxic natural compound, can trigger apoptosis in many cancer types. We now show that p53 is dispensable for Noscapine-induced cell death in neuroblastoma cell lines, proapoptotic response to this promising chemopreventive agent is mediated by suppression of survivin protein expression. The Noscapine treatment increased levels of total and Ser^15^-phosphorylated p53 protein in SK-SY5Y cells, but the proapoptotic response to this agent was maintained even after knockdown of the p53 protein level. Exposure of SK-SY5Y and LA1-5S cells to Noscapine resulted in a marked decrease in protein and mRNA level of survivin as early as 12 hours after treatment. Ectopic expression of survivin conferred statistically significant protection against Noscapine-mediated cytoplasmic histone-associated apoptotic DNA fragmentation. Also, the Noscapine-induced apoptosis was modestly but statistically significantly augmented by RNA interference of survivin in both cell lines. Furthermore, Noscapine-induced apoptotic cell death was associated with activation of caspase-3 and cleavage of PARP. In conclusion, the present study provides novel insight into the molecular circuitry of Noscapine-induced apoptosis to indicate suppression of survivin expression as a critical mediator of this process.

## Introduction

Neuroblastoma is a pediatric cancer of the developing sympathetic nervous system that most often affects young children. It remains an important pediatric problem because it accounts for approximately 15% of childhood cancer mortality [1]. Although improvement of multimodal therapy has been achieved by the progress of treatment, prognosis of this cancer, especially high-risk neuroblastoma, remains poor. On account of poor treatment outcome, new treatment strategies are constantly sought for neuroblastoma.

Among anticancer agents, antimicrotubules constitute one of the promising chemotherapeutic agents for treatment of different cancers. During cell division, α/β-tubulin polymerizes into dynamic structures called microtubules. Inhibitors of tubulin either target polymerization (vinca alkaloids and colchicine) or depolymerization (taxanes and epothilones). These two functional classes bind to different regions of the α/β-tubulin heterodimer and allosterically regulate tubulin oligomerization. Colchicine is deeply buried at the heterodimer interface and prevents a conformational change needed for polymerization [2], whereas taxanes bind in a shallow groove found on the β-subunit of tubulin in microtubules [3]. Small-molecule inhibitors of tubulin have been in clinical use since 1965. Many classes of tubulin inhibitors are known, and it appears that the presence of multiple allosteric binding sites makes tubulin particularly amenable to inhibition by small molecules [4]. Clinical use of currently available antimicrotubular agents has been limited due to drug-resistance, prolonged i.v. infusion and associated adverse side effects [5], [6]. Among antimicrotubule agents, noscapinoids constitute an emerging class of compounds receiving considerable attention for treating cancers due to oral available, improved patient compliance and minimal side effects compared to taxanes [7], [8], [9], [10]. Noscapine (Nos) attenuates microtubule dynamics just enough to activate the mitotic checkpoints to stop cell cycle and do not alter the steady state monomer/polymer ratio of tubulin [11] and demonstrated promising in vitro and in vivo antitumor activity against variety of cancers including resistant type [11], [12], [13], [14], [15], [16]. Based upon anticancer activity and non-toxic attributes, Noscapine is already in Phase I/II clinical trials.

We now show that p53 is dispensable for Noscapine-induced cell death, proapoptotic response to this promising chemopreventive agent is mediated by suppression of survivin protein expression. The Noscapine treatment increased levels of total and Ser^15^-phosphorylated p53 protein in NB cells, but the proapoptotic response to this agent was maintained even after knockdown of the p53 protein level. Exposure of SK-SY5Y and LA1-5S cells to Noscapine resulted in a marked decrease in protein level of survivin as early as 12 hours after treatment. Ectopic expression of survivin conferred statistically significant protection against Noscapine-mediated cytoplasmic histone-associated apoptotic DNA fragmentation in both cell lines, while knock-down of endogenous survivin by survivin siRNA sensitizes both cells to Noscapine-induced apoptosis. Furthermore, Noscapine-induced apoptotic cell death in SK-SY5Y and LA1-5S cells was associated with activation of caspase- 3 and cleavage of PARP. In conclusion, the present study provides novel insight into the molecular circuitry of Noscapine-induced apoptosis to indicate suppression of survivin expression as a critical mediator of this process.

## Results and Discussion

**Figure 1:**
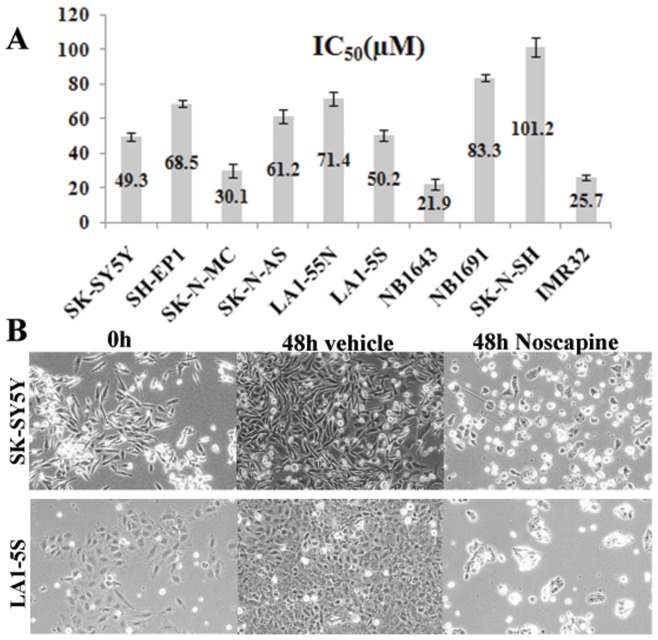
Noscapine suppresses proliferation of human neuroblastoma cells. **A**. A panel of Neuroblastoma cells (SK-SY5Y, SH-EP1, SK-N-MC, SK-N-AS, LA1-55N, LA1-5S, NB1643, NB1691, SK-N-SH and IMR32) were treated with gradient concentrations of Noscapine for 72 hrs and the IC50 values were evaluated by the sulforhodamine B (SRB) *in vitro* cell proliferation assay. The values and error bars shown in the graph represent average and standard deviations, respectively, of three independent experiments (p<0.05). **B**. Phase-contrast images (20×) of SK-SY5Y cells treated with 50 µM Noscapine for 0, and 48 hrs.

**Figure 2:**
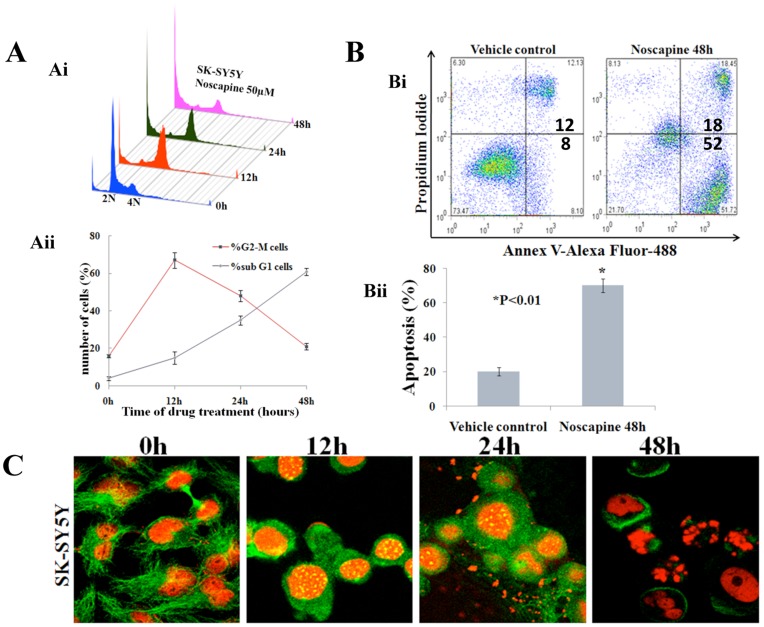
Noscapine perturbs cell-cycle progression of Sk-SY5Y cells and cause apoptosis. **A**. Effect of 25 µM EM011 on cell-cycle progression of A549 cells over time shown in a three-dimensional disposition. X-axis, intensity of propidium iodide fluorescence, is indicative of the total DNA content of cells in various phases of the cell-cycle. Y-axis, number of cells detected for a given DNA content. Z-axis, time points (i.e., 0, 12, 24, and 48 hrs). Representative results of three independent experiments. **B**. Quantitative graphical representation of the percentage of G2/M and sub-G1 cell population. Points, average of three independent experiments; bars, SD (p<0.05). C. Confocal immunofluorescence analyses of cells. Microtubules and DNA were visualized using fluorescent anti-alpha-tubulin antibody (green) and fluorescent DNA dye propidium iodide (red). SK-SY5Y cells begin to show mitotic arrest as early as 12 hours after drug treatment and begin to show disorganized arrest and chromatin decondensation or even death at 48 hours of treatment.

Noscapinoids represent a new generation of anticancer agents that modulate microtubule dynamics but do not significantly alter the total polymer mass of tubulin. To evaluate the efficacy of Noscapine in neuroblastoma cells, we first examined the ability of Noscapine to inhibit cellular proliferation in a comprehensive panel of neuroblastoma cells with variable but well-characterized genotypes. The panel included SK-SY5Y, SH-EP1, SK-N-MC, SK-N-AS, LA1-55N, LA1-5S, NB1643, NB1691, SK-N-SH and IMR32 neuroblastoma cells. These 10 different neuroblastoma cell lines were treated with gradient concentrations of Noscapine and the extent of cell proliferation was measured by the SRB assay, which is based on the stoichiometric binding of SRB dye to all cellular protein components [17]. As shown in Figure 1A, Noscapine effectively suppressed cellular proliferation of neuroblastoma cells. The neuroblastoma cell lines included in the study had well-characterized p53 status, namely, wild type (represented by SK-SY5Y, SH-EP1, NB1643, NB1691), null (LA1-55N, LA1-5S) and mutant (such as SK-N-AS). Although Noscapine has been shown to induce a p53-dependent apoptosis in colon cancer cells [18], these data show that the half-maximal growth inhibitory concentrations of Noscapine did not correlate with the p53 status in neuroblastoma cells. For example, LA1-5S that lacks endogenous p53 showed similar sensitivity as SK-SY5Y (wild type p53) or SK-N-AS (mutant p53) cells. The IC50 values for most neuroblastoma cell lines studied were in the range of 21–101 µM. The relatively less-sensitive neuroblastoma cells included SK-N-SH (101.2 µM), LA1-55N (IC50 = ∼81.4 µM) and SK-N-MC (IC50 =  ∼72.3 µM). We chose SK-SY5Y (wild type p53) and LA1-5S (p53null) for further studies to gain insights into cellular and molecular mechanisms of Noscapine action. Phase-contrast microscopic analysis of cell morphology showed that while vehicle-treated SK-SY-5Y (Figure 1B first panel) and LA1-5S (Figure 1B second panel) cells proliferated normally, Noscapine treatment impaired their proliferation capacity. Cells first appeared rounded-up (12 hrs and 24 hrs treatment (Figure 2C), followed by a fragmented morphology (48 hrs treatment, Figure 1B, 2C).

To investigate the precise mechanism responsible for Nos-mediated antiproliferative effects, we examined the cell-cycle distribution profile of Nos-treated SK-SY5Y neuroblastoma cells over time. A flow-cytometric assay using the DNA intercalator dye, propidium iodide (PI), was utilized to monitor cell-cycle progression on the basis of status of DNA amounts. Figure 2Ai shows the time dependent effects of Noscapine on cell-cycle profile of SK-SY5Y cells in a three-dimensional representation. The x-axis shows amount of DNA depicting different phases of the cell-cycle. While 2N and 4N DNA complements represent G0/G1 and G2/M cell populations respectively, S phase is characterized by variable DNA (between 2N and 4N) and sub-G1 population is usually indicative of degraded DNA, a hallmark of apoptosis. The y-axis represents the number of cells containing that amount of DNA and the z-axis shows the time of drug-exposure. 0 hr depicts cell-cycle profile of control cells. As we go along in time, Noscapine treatment caused a significant inhibition of cell-cycle progression in SK-SY5Y cells resulting in an accumulation of cells in the G2/M phase compared to control cells (Figure 2Ai).The G2/M population achieved a maximum at 12 hrs (∼67%) and was still about ∼48% at 48 hrs of Noscapine exposure (Figure 2Aii). This increased population of cells with 4N DNA perhaps correlated with concomitant losses from G0/G1 phases (Figure 2Ai). Following this, a disappearance of the G2/M population and an emergence of a characteristic hypodiploid DNA content peak (sub-G1) was observed beginning 12 hrs, indicative of apoptotic cells (Figure 2Ai). The sub-G1 population at 12 hrs (∼15%) increased to ∼61% at 48 hrs post-treatment (Figure 2Ai).

Fluorecent-activated sell sorting (FACS) analyses of DNA content showed a decline in G1 and a concomitant rise in G2/M. It also showed a distinct shift of cells to a sub-G1 DNA content of <2 N at 48 h Noscapine-treatment suggesting DNA-degredation associated with apoptosis (Figure 2Aii). Careful confocal microscopic examination of the MT arrays and the chromosomes in drug-treated cells revealed a typical mitotic arrest with bipolar prometaphase spindles with tightly condensed chromosomes. In contrast, highly disorganized, often multipolar mitoses were visible with less condensed chromosomes and appeared to die 48-hours post treatment (Figure 2C).

To determine the extent of the apoptotic onset caused by Noscapine in SK-SY5Y cells, we analyzed the early apoptotic cells that externalize the normally internal membrane lipid, phosphatidylserine (PS), by binding to a fluorescently conjugated PS-binding protein, Annexin V-Alexa Fluor488. These cells can be distinguished from the late apoptotic cells that also allow the DNA dye, propidium iodide (PI) to penetrate the cell membranes allowing intracellular DNA to bind it. As shown in Figure 2B, 70.1% cells were in early and late stages of apoptosis after a 48 hour Noscapine treatment (50 µM) as compare to a modest 20.2% at vehicle control treatment (Figure 2Bi, Bii). The same results were also confirmed in LA1-5S cell lines (data not shown). We conclude that Noscapine caused programmed cell death both in SK-SY5Y and LA1-5S neuroblastoma cell lines.

**Figure 3:**
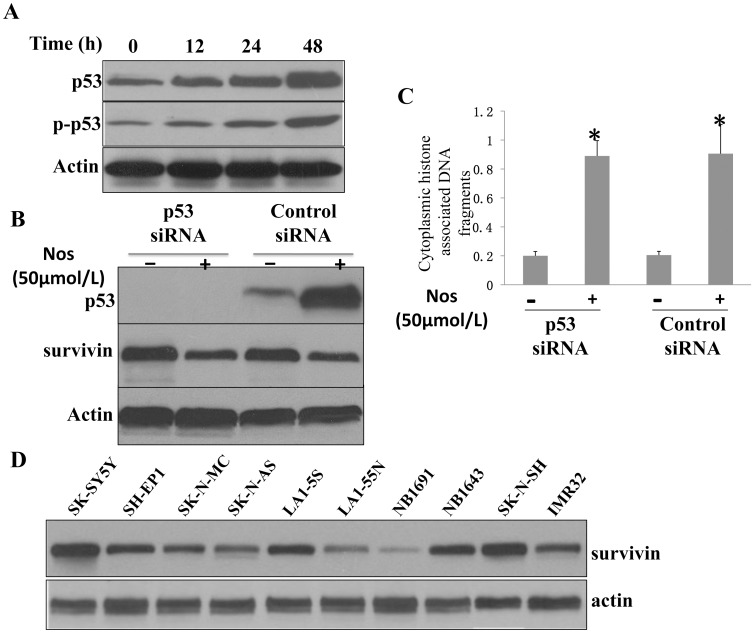
Noscapine cause p53 independent survivin downregulation. A, immunoblotting for total and phospho-(Ser^15^)-p53 using lysates from SK-SY5Y cells treated with DMSO (control) or Noscapine (50 µmol/L) for the indicated time periods. B, immunoblotting for p53 using lysates from SK-SY5Y cells transfected with a control nonspecific siRNA or a p53-targeted siRNA and treated for 24 h with DMSO (control) or 50 µmol/L Noscapine. C, cytoplasmic histone-associated apoptotic DNA fragmentation in SK-SY5Y cells transiently transfected with a control nonspecific siRNA or a p53-targeted siRNA and treated for 24 h with DMSO (control) or 50 µmol/L Noscapine. Columns, mean (n = 3); bars, SD. *, significantly different (P<0.05) compared with nonspecific siRNA-transfected cells treated with DMSO by one-way ANOVA followed by Bonferroni’s test. The results were consistent in three independent experiments, and representative data from one such experiment are shown. D, Immunoblotting for survivin using lysates from a series of NB cell lines.

We used the same cell line to test whether Noscapine-induced apoptosis was influenced by p53, which is a well-accepted facilitator of apoptotic cell death by different stimuli [19]. As can be seen in Figure 3A, Noscapine exposure caused a time-dependent increase in the levels of total as well as Ser^15^-phosphorylated p53. The Ser^15^ phosphorylation of p53 has been implicated in apoptosis [19]. Next, we used siRNA technology to directly test possible involvement of p53 in regulation of Noscapine-induced apoptosis. Transient transfection of SK-SY5Y cells with a p53-targeted siRNA resulted in complete silencing of the p53 protein expression (Figure 3B). Moreover, the Noscapine-mediated induction of p53 protein was abolished in SK-SY5Y cells transfected with the p53-specific siRNA (Figure 3B). However, enrichment of cytoplasmic histone-associated apoptotic DNA fragmentation resulting from Noscapine exposure (50 µmol/L for 24 h) over DMSO-treated control was comparable in SK-SY5Y cells transfected with the control nonspecific siRNA and p53-targeted siRNA (Figure 3C). Knock-down of p53 using p53 siRNA didn’t alter the apoptotic response of SK-SY5Y cells to Noscapine treatment. Collectively, these results indicated that the Noscapine-induced apoptosis was not influenced by the p53 status at least in the SK-SY5Y cell line.

**Figure 4:**
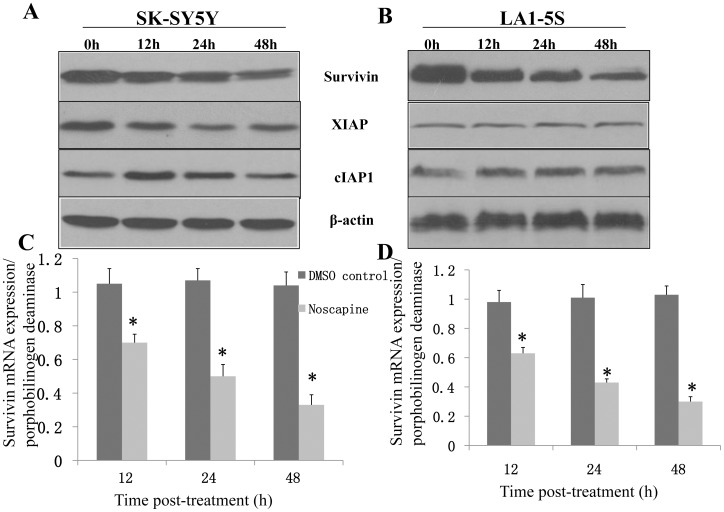
Noscapine selectively cause survivin downregulation in SK-SY5Y and LA1-5S cells. Immunoblotting for survivin, cIAP1and XIAP using lysates from SK-SY5Y(A) and LA1-5S (B) cells treated with DMSO (control) or Noscapine (50 µmol/L) for the indicated time periods. Immunoblotting for each protein was done at least twice using independently prepared lysates. Representative data from one such experiment are shown. Histograms represent the increase or decrease of survivin mRNA expression in SK-SY5Y (C) and LA1-5S (D) cells incubated with noscapine (50 µM/mL) for 12, 24, and 48h. Measurements were performed in triplicate. Bars represent standard errors and asterisks designate statistically significant results (*P*<0.05).

Since knockdown of p53 had no influence on proapoptotic response to Noscapine in SK-SY5Y cells, IAP proteins, including cIAP1, XIAP, and survivin have emerged as critical regulators of apoptotic cell death [20]–[21], we raised the question of whether the Noscapine induced apoptosis in neuroblastoma cells was accompanied by alterations in expression of IAP family proteins. The IAPs play important roles in adaptive response to cellular stress, differentiation, motility, and immune response [22]. This family of proteins is characterized by the presence of baculovirus IAP repeat (BIR) domains [23]. The survivin protein expression was markedly decreased on treatment of SK-SY5Y and LA1-5S cells with Noscapine at 50 µmol/L concentrations. The Noscapine-mediated decline in survivin protein level was evident as early as 12 hours after treatment, and this effect was maintained for the duration of the experiment (48 h after treatment) in both cell lines (Figure 4). The level of cIAP1 protein was increased on Noscapine treatment in both SK-SY5Y and LA1-5S cell lines, but this effect was not sustainable (Figure 4). For example, Noscapine-mediated induction of cIAP1 protein was nearly completely abolished at the 48-hour time point in the SK-SY5Y cell line. On the other hand, the Noscapine treatment did not have an appreciable effect on XIAP protein level both in SK-SY5Y and LA1-5S cell lines. We also test the level of survivin in p53 knochout experiment. As shown in figure 3B, survivin levels didn’t have apparent changes whether p53 was knockout or not, but do have changes when treated with Noscapine. Collectively, these results indicated that Noscapine treatment caused a sustained decrease in the level of survivin protein in both cell lines.

Survivin, a 17 kD protein that contains only a single BIR and no RING dormain, is the smallest member of IAPs and stands out for its clear association with cancer [24], [25]. Reactivation of the survivin gene occurs frequently during tumorigenesis and in most cases correlates with drugs resistance and shorter disease free and poor overall survival [26], [27]. Synthesis and degradation of survivin are modulated in a cell cycle-dependent manner. Transcription increases during G_1_ and reaches a peak at G_2_/M, which is controlled by specific sequences in the promoter region involving Sp1 and cell cycle-dependent element binding sites [28]. One of the first characteristics of survivin to be described was its ability to bind to microtubules through the carboxy-terminal coiled-coil dormain, and to assist in chromosomal segregation and cytokinesis during mitosis [29], [30].

**Figure 5:**
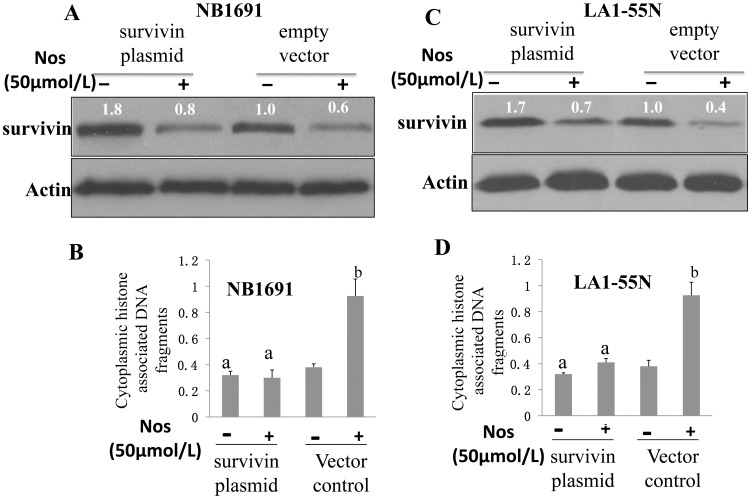
Overexpression of survivin conferred protection Noscapine-induced apoptosis. Noscapine-inducedapoptosis.Immunoblotting for survivin protein using lysates from NB1691(A) and LA1-55N (C) cells transiently transfected with empty pcDNA3.1 vector or pcDNA3.1 vector encoding for survivin and treated for 24 h with DMSO (control) or 50 µmol/L Noscapine. The numbers above the immunoreactive bands represent change in protein level relative to empty vector–transfected cells treated with DMSO (lane 1). Cytoplasmic histone-associated apoptotic DNA fragmentation in NB1691 (B) and LA1-55N (D) cells transiently transfected with empty pcDNA3.1 vector or vector encoding for survivin and treated for 24 h with DMSO (control) or 50 µmol/L Noscapine. Columns, mean (n = 3); bars, SD. Significantly different (P<0.05) compared with empty vector–transfected cells treated with DMSO (a) and empty vector–transfected cells treated with Noscapine (b) by one-way ANOVA followed by Bonferroni’s test. Comparable results were observed in three independent experiments. Representative data from one such experiment are shown.

Although pioneering studies of Hoffman and colleagues [31], and Mirza and colleagues [32] identified survivin as one of the relatively few known genes that is actively repressed by wild type p53, we failed to identify this effectiveness in SK-SY5Y cell line which with wilt type p53. After deletion of p53 with siRNA in SK-SY5Ycells, the survivin protein was not apparantly changed (data not shown). To reveal the precise mechanism by which Noscapine treatment suppresses expression of survivin protein, we have had carefully examination of the survivin mRNA using rt-PCR and a significant down-regulation (of 35∼70% in SK-Y5Y and 30∼75% in LA1-5S) of survivin mRNA expression were observed at 12, 24 and 48 h post-treatment (P<0.05) (Figure 4C, 4D). Because Noscapine treatment exhibited the most striking effect on survivin protein as well as mRNA expression in both cell lines (Figure 4), we designed experiments to determine functional significance of these observations. After screening all the neuroblastoma cell lines in our lab, we found notable difference between the cell lines (figure 3D). We use another two cell lines (NB1691 and LA1-55N) that have least basal level of survivin for purpose of further over expression experiment. Transient transfection of NB1691 cells with a vector encoding for survivin resulted in ∼1.8-fold increase in its protein level compared with empty vector–transfected cells (Figure 5A). The Noscapine treatment (50 µmol/L for 24 h) caused a decrease in the level of survivin protein both in empty vector–transfected NB1691 cells and in survivin -overexpressing cells (Figure 5A). In addition, overexpression of survivin conferred significant protection against Noscapine-induced cytoplasmic histoneassociated DNA fragmentation in NB1691 cells (Figure 5B). Statistically significant inhibition of Noscapine-induced cytoplasmic histone-associated DNA fragmentation by forced overexpression of survivin was also observed in the LA1-55N cell line (Figure 5C, 5D). These results indicated that survivin was a target of Noscapine-induced apoptosis in neuroblastoma cells, at least in NB1691 and LA1-55N cell lines.

**Figure 6:**
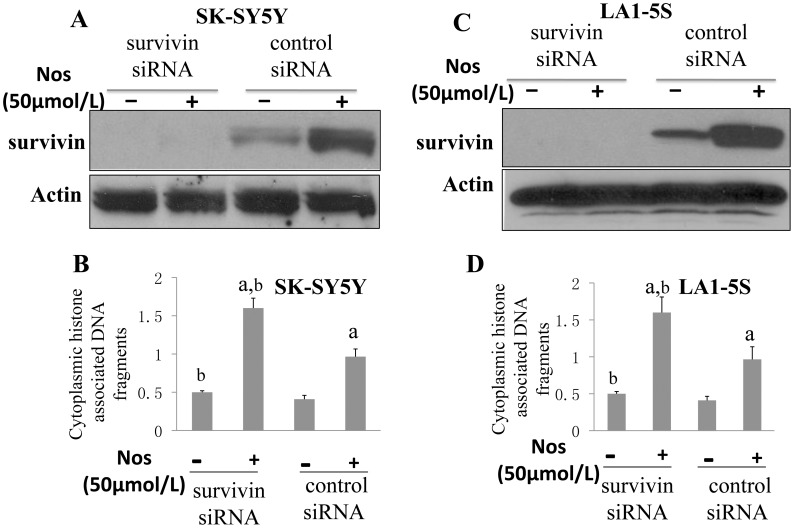
Knock-down endogenous survivin render SK-SY5Y and LA1-5S cells more susceptible to Noscapine. Immunoblotting for survivin using lysates from SK-SY5Y (A) and LA1-5S (C) cells transiently transfected with a control nonspecific siRNA or a survivin-specific siRNA and treated with DMSO (control) or 50 µmol/L Noscapine for 24 h. Cytoplasmic histone-associated DNA fragmentation in SK-SY5Y (B) and LA1-5S (D) cells transiently transfected with a control nonspecific siRNA or a survivin-specific siRNA and treated with DMSO (control) or 50 µmol/L Noscapine for 24 h. Columns, mean (n = 3); bars, SD. Significantly different (P<0.05) compared with control nonspecific siRNA-transfected cells treated with DMSO (a) and control nonspecific siRNA-transfected cells treated with Noscapine (b) by one-way ANOVA followed by Bonferroni’s test. Results were consistent in two experiments, and representative data from one such experiment are shown.

If survivin is important to Noscapine-induced apoptosis, knock-down of endogenous survivin expression should render SK-SY5Y and LA1-5S cells to become more susceptible to Noscapine treatment. Thus, to determine whether downregulation of survivin contributes to Noscapine-induced apoptosis, we used a plasmid construct encoding survivin specific siRNA to selectively knock-down endogenous survivin in SK-SY5Y and LA1-5S cells. Exposure of nonspecific siRNA-transfected SK-SY5Y cells to 50 µmol/L Noscapine for 24 hours resulted in ∼3-fold increase in survivin protein level compared with DMSO-treated control (Figure 6A). The level of survivin protein was decreased by >95% in SK-SY5Y cells transfected with a survivin-targeted siRNA (Figure 6A). Moreover, the Noscapine-mediated induction of survivin protein expression was nearly completely abolished in survivin siRNA transfected cells. As shown in Figure 6B, the cytoplasmic histone-associated DNA fragmentation enrichment resulting from Noscapine exposure over DMSO-treated control was modestly but statistically significantly greater in survivin silenced cells in comparison with nonspecific siRNA transfected SK-SY5Y cells (Figure 6B). On the other hand, knockdown of survivin protein level (Figure 6C) also have an appreciable effect on Noscapine-induced apoptosis in the LA1-5S cell line (Figure 6D). We conclude that induction of survivin is marginally cytoprotective against Noscapine-mediated cell death in the SK-SY5Y and LA1-5S cells.

**Figure 7:**
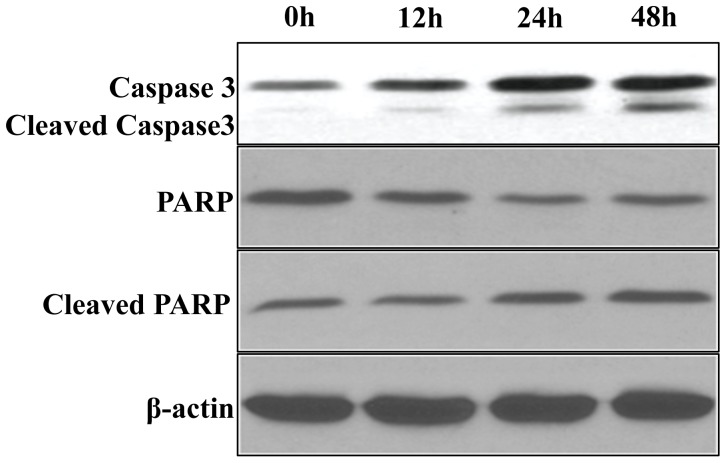
Noscapine induced apoptosis via activation of caspase- 3 and PARP cleavage. Immunoblot analysis of SK-SY5Y and LA1-5S cells treated with noscapine for 0, 12, 24 and 48 hrs. After the indicated times, cells were lysed and total protein was extracted, separated by SDS-PAGE, electrotransferred onto polyvinylidene difluoride membrane, and subjected to immunoblotting with the indicated primary antibodies followed by incubation with horseradish peroxidase- conjugated secondary antibodies. β-actin was used as a loading control.

Upon cleavage by upstream proteases in an intracellular cascade, the activation of caspase-3 is considered as a hallmark of the apoptotic process. The levels of cleaved active subunits of executioner caspase-3 were evaluated by immunoblotting cell lysates following Noscapine treatment for 0, 12, 24, and 48 hrs. Noscapine caused a significant increase in activated caspase-3 following 48 hrs of Noscapine exposure (Figure 7). To confirm the involvement of caspase-3, the active form of the cysteine protease was monitored using a small conserved modified peptide substrate that becomes luminogenic upon cleavage. Noscapine treatment caused a time-dependent activation of caspase-3 in SK-SY5Y cells (Figure 7). Next, we examined the activation mediated cleavage of caspase-3 substrate, poly(ADPribose) polymerase (PARP), which is a reliable marker of apoptosis. Utilizing their cysteine protease activity, caspases separate N-terminal DNA-binding domain of PARP from its C-terminal catalytic domain (89 kDa) [33]. A time-dependent increase in cleaved PARP was observed upon probing with a cleaved PARP specific antibody (Figure 7). Overall, these results show activation of caspase- 3 and PARP cleavage suggesting that Noscapine induced apoptotic cell death in SK-SY5Y cells. Same results were also observed in LA1-5S cell line (data not shown).

In conclusion, we provide experimental evidence to show that the Noscapine-induced apoptosis in human neuroblastoma cells is independent of p53 but mediated by suppression of survivin protein level. The cell death program is mediated through downregulaion of survivin, in that knock-down of survivin sensitized cells to undergo apoptosis whereas overexpression of survivin reduced Noscapine-induced apoptosis. The inhibition of cellular proliferation is perhaps due to induction of a robust transient mitotic arrest. This is followed by activating caspase machinery by the cleavage of downstream targets such as PARP. This study provides evidence for the potential usefulness of Noscapine, a non-toxic microtubule-modulating agent, in chemotherapy of neuroblasctoma.

## Materials and Methods

### Chemicals and Reagents

Noscapine, Sulforhodamine B (SRB), 4-6-diamidino-2-phenylindole (DAPI), propidium iodide (PI), RNase, bovine serum albumin (BSA), and the mouse monoclonal antibody against α-tubulin were from Sigma-Aldrich (St. Louis, MO, US). anti-p53 antibody was from Calbiochem; antibodies against cIAP1, phospho-p53 (Ser^15^), caspase-3, cleaved PARP were obtained from Cell Signaling (Beverly, MA); anti-survivin antibody was from Novus Biologicals; antibody against X-linked inhibitor of apoptosis (XIAP) was from BD Biosciences. Horseradish peroxidaseconjugated anti-rabbit and anti-mouse secondary antibodies were purchased from Sigma. Fluorescein-conjugated anti-mouse antibody was from Jackson ImmunoResearch, Inc. (West Grove, PA). RPMI 1640 and MEM were purchased from Cellgro. The p53- and survivin-targeted small interfering RNA (siRNA) were purchased from Santa Cruz Biotechnology. A nonspecific control siRNA was from Qiagen. FuGENE6 transfection reagent and a kit for quantification of cytoplasmic histone-associated apoptotic DNA fragmentation were procured from Roche Applied Sciences.

### Cell Lines and Plasmids

Human neuroblastoma cells (SK-SY5Y, SH-EP1, SK-N-MC, SK-N-AS, LA1-55N, LA1-5S, NB1643, NB1691, SK-N-SH and IMR32) were well established cell lines [34], [35], [36], [37], [38] and obtained from Dr. Muxiang Zhou’s laboratory, Children oncology/hematology, Emory University. All cells were maintained in RPMI-1640 media supplemented with 10% fetal bovine serum (Invitrogen Carlsbad, CA) and 1% antibiotics. The plasmid vector with survivin siRNA sequence 5′-GGCTGGCTTCATCCACTGCCC-3′was generated by cloning the synthesized oligonucleotide into pSilencer 2.1-U6Neo plasmid (Ambion Inc., Austin, TX). Control pSilencer 2.1- U6 Neo plasmid vector containing a scrambled siRNA sequence, 5′-ACTACCGTTGTTATAGGTGT-3′, was obtained from Ambion Inc. The survivin plasmid contruct was cloned into the pcDNA3 vector and pcDNA3 vector alone served as control. All transfections were done using Lipofectamine 2000 following the manufacturers’ instructions.

### *In vitro* Cell Proliferation Assay

Neuroblastoma cells were seeded in 96-well plates at a density of 5×10^3^ cells/well followed by next day treatment with increasing gradient concentrations of Noscapine ranging from 10 nm to 100 mM. After 48 hrs of drug treatment, cells were fixed with 50% trichloroacetic acid and stained with 0.4% sulforhodamine B (SRB) dissolved in 1% acetic acid. Cells were then washed with 1% acetic acid to remove the unbound dye. Essentially, the SRB assay measures cell density by quantitating colored SRB bound to cellular proteins fixed to the plates by tricholoroacetate. The protein-bound dye was extracted with 10 mM Tris-base to determine the absorbance at 564 nm wavelength [17], [39]. The percentage of cell survival as a function of drug concentration was then plotted to determine IC50 values (drug concentration needed to prevent cell proliferation by 50%).

### Cell-cycle Analysis

SK-SY5Y and LA1-5S cells were seeded in culture dishes and grown until ∼70% confluence. The medium was then replaced with new medium containing either vehicle (0.01% DMSO) or 25 µM Noscapine for 6, 12, 18, 24, 36 and 48 hrs. After the incubation period, cells were centrifuged, washed twice with ice-cold PBS, and fixed in 70% ethanol. Tubes containing the cell pellets were stored at 4°C for at least 24 hrs. Cells were then centrifuged at 100×g for 10 minutes, and the supernatant was discarded. Pellets were washed twice with PBS and stained with PI in the presence of RNase A for 45 minutes in dark. Samples were analyzed on a FACSCalibur flow-cytometer (Beckman Coulter, Inc., Fullerton, CA).

### Immunofluorescence Confocal Microscopy

SK-SY5Y and LA1-5S cells were grown on poly(L-lysine)-coated glass coverslips for immunofluorescence microscopy as described previously [10], [40]. After treatment, cells were fixed with cold (−20°C) methanol for 5 minutes and blocked by incubating with 2% BSA/PBS at 37°C for 1 hr. A mouse monoclonal antibody against α-tubulin (DM1A, Sigma) was diluted 1∶500 in 2% BSA/PBS and incubated with the coverslips for 2 hrs at 37°C. Cells were then washed with 2% BSA/PBS for 10 minutes at room temperature before incubating with a 1∶200 dilution of a FITC-labeled goat anti-mouse IgG antibody (Jackson ImmunoResearch, Inc., West Grove, PA) at 37°C for 1 hr. Coverslips were then rinsed with 2% BSA/PBS for 10 minutes and incubated with PI (0.5 µg/mL) for 15 minutes at room temperature before they were mounted with Aquamount (Lerner Laboratories, Pittsburgh, PA) containing 0.01% 1,4-diazobicyclo(2,2,2)octane (Sigma). Cells were examined using confocal microscopy for microtubule and nuclear morphology using a 63× objective (numerical aperture, 1.4).

### Western Blot Analysis

Proteins were resolved by polyacrylamide gel-electrophoresis and transferred onto polyvinylidene difluoride membranes (Millipore). Membranes were blocked in Trisbuffered saline containing 0.2% Tween-20 and 5% fatfree dry milk and incubated first with primary antibodies and then with horseradish peroxidase-conjugated secondary antibodies. Specific proteins were visualized with enhanced chemiluminescence detection reagent according to the manufacturer’s instructions (Pierce Biotechnology).

### Quantitative Real-time Reverse Transcriptase-polymerase Chain Reaction

Total cellular RNA was extracted from SK-SY5Y and LA1-5S cells using Trizol reagent (Gibco) according to the manufacturer’s instructions. Preservation of 28S and 18S rRNA species was used to assess RNA integrity. Only samples with prominent 28S and 18S rRNA components were included in the study. Of the total RNA, 1 µg from each sample was reverse transcribed to complementary DNA (cDNA) using Moloney Murine Leukemia Virus reverse transcriptase (Invitrogen, Life Technologies, Paisley, UK) and random primers (Invitrogen). Survivin mRNA expression was evaluated by real-time polymerase chain reaction using FastStart Universal SYBR Green Master (ROX) (Roche, Mannheim, Germany) in a iCycler Optical Module (Bio-Rad, Hercules, CA, USA). Reactions were performed in triplicate using 2 µL of cDNA per reaction and primers specific for survivin (forward: 5′ CGA GGC TGG CTT CAT CCA 3′; reverse: 5′ GCA ACC GGA CGA ATG CTT T 3′′), while human porphobilinogen deaminase was used as a housekeeping gene (forward: 5′ AGA GTG ATT CGC GTG GGT ACC 3′; reverse: 5′ GGC TCC GAT GGT GAA GCC 3′).

### Gene Silencing with Small Interfering RNAs

siRNA oligonucleotides were purchased from Ambion/Applied Biosystems (Austin, TX). All siRNAs were reconstituted according to the manufacturer’s instructions. Briefly, The SK-SY5Y and LA1-5S cells were seeded in six-well plates and transfected at ∼50% confluency with 100 nmol/L of p53- or survivin-targeted siRNA using Oligofectamine. Twenty-four hours after transfection, the cells were treated for 24 hours with either DMSO (control) or 50 µmol/L Noscapine. Subsequently, the cells were collected and processed for immunoblotting and flow cytometric analysis.

### Ectopic Expression of Survivin by Transient Transfection

The SK-SY5Y and LA1-5S cells were transiently transfected at ∼50% confluency with the empty pcDNA3.1 vector or pcDNA3.1 encoding for survivin (Addgene) using FuGENE6 transfection reagent. Twentyfour hours after transfection, the cells were treated with DMSO (control) or Noscapine for specified time period. Cells were collected and processed for immunoblotting and measurement for apoptosis.

### Statistical Analysis

Each experiment was repeated at least twice with triplicate measurements for quantitative comparisons. Statistical significance of difference in measured variables between control and treated groups was determined by t test or one-way ANOVA. Difference was considered significant at P<0.05.
